# Violence prevention: the case for action

**DOI:** 10.2471/BLT.22.288721

**Published:** 2022-07-01

**Authors:** Kidist Kebede Bartolomeos

**Affiliations:** a Quality Assurance, Norms and Standards Department, World Health Organization, Avenue Appia 20, 1211 Geneva 27, Switzerland.

The World Health Organization (WHO) defines violence as “the intentional use of physical force or power, threatened or actual, against oneself, another person, or against a group or community, that either results in or has a high likelihood of resulting in injury, death, psychological harm, maldevelopment, or deprivation.”^1^ WHO estimates that violence affects the lives of up to 1 billion children globally and that it has long-lasting and costly emotional, social and economic consequences.^2^ The sustainable development goal (SDG) target 16.2 calls for the protection and realization of the right of every child to live free from fear, neglect, abuse and exploitation, and calls to end all forms of violence.^3^ However, according to a 2020 self-reported survey, WHO Member States are not taking sufficient steps to achieve SDG target 16.2.^2^

The same week that Member States gathered in Geneva, Switzerland to discuss the Global Health for Peace Initiative,^4^ in the United States of America, gun violence forever changed individuals, families and the community in Uvalde, Texas when a gunman killed 19 children and two adults at an elementary school.^5^ Mass shootings such as Uvalde’s are not isolated incidents. Violence-related injuries and deaths are common daily events in many parts of the world. WHO’s landmark 2002 report estimated that each year, about 1 million people lost their lives due to self-inflicted, interpersonal or collective violence.^1^ Those estimates are not much different today. Most violent deaths occur outside of conflict settings, in homes, streets and other public settings.^6^ Every day, children and young adults lose their life due to violence. Those who survive might have lifelong physical and mental disabilities due to their injuries, as well as experience other consequences such as financial and social costs. Injuries due to interpersonal violence and self-harm are among the leading causes of death in older children and adolescents.^7^

These deaths and injuries affect people and communities, causing psychological and emotional trauma, social tensions and insecurity. As public health professionals, we need to demand from policy-makers sustainable solutions to address the root causes of all forms of violence including gun violence. Inaction is not an option. Several reasons exist for moving from analysis to action. 

First, we must act because as individuals and as a scientific, global public health community, we have a moral and professional obligation to advocate for change. WHO defines health as “a state of complete physical, mental and social well-being and not merely the absence of disease or infirmity.”^7^ WHO’s constitution states that enjoyment of the highest attainable standard of health is one of the fundamental rights of every human being without distinction of race, religion, political belief, economic or social condition.^8^ Children, regardless of where they live, need our protection and advocacy for their health, well-being and safety.

Second, we must act because when an individual’s action is meant to cause harm, even one incident is one too many. According to a 2016 estimate, between 195 000 and 276 000 firearm-related injury deaths occur globally each year, most of which are homicides.^9^ Another study suggests that the level of gun violence varies among countries and across demographic subgroups, but that gun violence is an issue in all countries, regardless of their income level.^9^ The 2020 WHO report on violence against children alerted that even in countries with violence-prevention laws, an enforcement gap exists.^2^

Finally, we must act because violence, like health conditions, can be analysed and addressed using data, evidence and science to inform policy and advocate for change. The high burden and impact of violence-related injuries and violence on society makes violence a public health issue. The behavioural, social, economic and political factors contributing to violence can be studied to inform policy change; therefore, violence is a preventable public health issue. In addition, measures such as mental health and psychosocial support for victims of violence, and improved emergency response can be put in place to minimize its impact.

The recent event in Uvalde is another reminder of the cost of inaction. Public health advocates have long been calling on governments to address violence, including violence against children, as a public policy issue as well as a public health issue. We need to use every opportunity we have to call on policy-makers to act. Violence prevention can be a means of promoting health, dignity, safety and overall peace within our communities. However, doing so requires that our elected officials fulfil their obligation to put the communities and nations they are elected to serve first, and take the lead in violence prevention. 
